# Evolution of infectious bronchitis virus in the field after homologous vaccination introduction

**DOI:** 10.1186/s13567-019-0713-4

**Published:** 2019-11-09

**Authors:** Giovanni Franzo, Matteo Legnardi, Claudia Maria Tucciarone, Michele Drigo, Marco Martini, Mattia Cecchinato

**Affiliations:** Department of Animal Medicine, Production and Health (MAPS), University of Padua, Viale dell’Università 16, 35020 Legnaro, PD Italy

## Abstract

Despite the fact that vaccine resistance has been typically considered a rare phenomenon, some episodes of vaccine failure have been reported with increasing frequency in intensively-raised livestock. Infectious bronchitis virus (IBV) is a widespread avian coronavirus, whose control relies mainly on extensive vaccine administration. Unfortunately, the continuous emergence of new vaccine-immunity escaping variants prompts the development of new vaccines. In the present work, a molecular epidemiology study was performed to evaluate the potential role of homologous vaccination in driving IBV evolution. This was undertaken by assessing IBV viral RNA sequences from the ORF encoding the S1 portion of viral surface glycoprotein (S) before and after the introduction of a new live vaccine on broiler farms in northern-Italy. The results of several biostatistics analyses consistently demonstrate the presence of a higher pressure in the post-vaccination period. Natural selection was detected essentially on sites located on the protein surface, within or nearby domains involved in viral attachment or related functions. This evidence strongly supports the action of vaccine-induced immunity in conditioning viral evolution, potentially leading to the emergence of new vaccine-escape variants. The great plasticity of rapidly-evolving RNA-viruses in response to human intervention, which extends beyond the poultry industry, is demonstrated, claiming further attention due to their relevance for animal and especially human health.

## Introduction

Avian infectious bronchitis is a well-recognized and widespread disease, which entails remarkable economic losses to the poultry industry by inducing respiratory and reproductive signs, decreased productive performances and increased mortality, particularly when nephropathogenic strains or secondary infections are involved [1].

The etiological agent, avian infectious bronchitis virus (IBV), is a member of the species *avian Coronavirus*, order *Nidovirales*, suborder *Cornidovirineae*, family *Coronaviridae*, subfamily *Orthocoronavirinae*, genus *Gammacoronavirus*, subgenus *Igacovirus* [2]. The viral genome is about 27 kb long and encodes non-structural (such as the RNA-dependent RNA polymerase (RdRp) and other accessory and regulatory proteins) and structural proteins (i.e., the spike, envelope, membrane, and nucleocapsid) [3]. Among the others, the spike protein (S), and the sub-unit S1 in particular, is widely studied because of its role in cell tropism, receptor attachment, neutralizing antibodies and cell-mediated immune response induction [1, 4, 5]. Additionally, the high genetic variability of the S1 gene has prompted its use for the classification of IBV strains into genotypes and lineages, which can display significantly different geographical distribution and, in some instances, biological behavior and immunological features [6].

In fact, as other positive-sense single-stranded RNA viruses, IBV is able to evolve rapidly and to recombine [1, 7, 8], leading to the emergence of a remarkable genetic and phenotypic variability over time. This heterogeneity poses noteworthy challenges to the understanding of IBV epidemiology and its control. Currently, vaccination represents the most applied and effective strategy to limit the disease impact. Nevertheless, the antigenic variability entails the existence of several serotypes and protectotypes, which translate in a poor cross-protection among genotypes [9], requiring the use of different vaccine combinations in order to broaden the protection spectrum or the development of new vaccines against recently emerged or introduced genotypes [10, 11]. Unfortunately, even closely related vaccines can fall into episodes of incomplete protection or vaccine immune-escape because of amino acid substitution in specific antigenic sites [9].

Despite the quite clear scenario, the understanding of underlying forces prompting the viral phenotypic variability is much more nuanced.

A high mutation rate does not automatically lead to a comparably elevated heterogeneity in biological features: the persistence and spread of new phenotypic variants implies that they must be favorably selected by the environment [12]. Although different kinds of mutations can alter the viral behavior and biology, the occurrence of non-synonymous mutations in relevant protein regions is probably the most recognized and studied evolutionary mechanism.

In this sense, the host population immunity represents one of the most obvious forces that can promote viral diversification, especially in antigenic regions. Besides natural immunity, vaccine administration could significantly contribute to this process. The role of vaccination in driving viral evolution has been reported for different diseases affecting both animals and human beings. When immunity is not sterilizing, wild strains are able to circulate in a new and more challenging environment, potentially adapting to it [13, 14]. Numerous examples can be mentioned for viruses that, to different degrees, circumvented vaccination strategies by immuno-escape (Hepatitis B virus, avian Metapneumovirus, Porcine circovirus type 2) [15–17], virulence increase (Marek disease virus) [18], or both (Infectious bursal disease virus) [18].

Nevertheless, the confirmation of wide vaccination application as a driver of IBV evolution is challenging. Particularly, the lack of systematic studies and the application of different vaccination protocols in different farms create a multitude of confounding effects hindering the identification of the actual underlying biological phenomena.

The present study investigates the impact of the homologous vaccination application on the evolution of IBV QX genotype (GI-19 lineage) S1 subunit in field conditions, circumventing the above-mentioned limitations and benefiting of the peculiar Italian epidemiological scenario and related control strategies applied in response to QX introduction in this country.

The proposed model was selected and considered extremely favorable for several reasons:

1. The QX genotype, and IBV in general, displays a high mutation rate (i.e. approximatively 10^−3^–10^−5^ substitutions/site/year) and therefore a relevant mutation number is expected to accumulate even over a relatively short time period [19].
2. The immunological features of IBV have been extensively studied. Particularly, the spike protein has been recognized as the most relevant target of the host immune response [4, 20]. Therefore, a S1-focused study design, limiting the technical problems and maximizing the biological information can be defined.
3. GI-19 is one of the most relevant lineages in the world, and the most significant one in Italy. Our previous studies have provided a detailed characterization of its molecular epidemiology in Italy and integrated this information with the remarkable, and ever increasing, number of sequences released worldwide [19, 21, 22]. This bulk of data allowed to demonstrate that after the introduction in Italy, QX strains had a substantially independent evolution [19].
4. The Italian poultry industry is dominated by few integrated companies, mainly located in northern Italy. Consequently, the applied control strategies are highly homogeneous among farms. Particularly, a homologous QX vaccine has recently been introduced by some of these companies and applied to all affiliated broiler farms, replacing a Mass + 793B based vaccination. Therefore, a clear comparison between the pre- and post-homologous vaccination application was possible.

## Materials and methods

### Sample collection and sequencing

Four hundred and ten samples (pools of ten tracheal swabs), previously tested IBV positive using the Virus-IBV-kit (Gensig, Southampton, UK), were collected between 2012 and 2017 from broiler farms located in northern Italy and delivered to the laboratory of infectious diseases of the Dept. Animal medicine, Production and Health (Padua University) for sequencing and genotyping. An IBV hypervariable region was amplified using the XCE1 and XCE2 primers as described by Cavanagh et al. [23] and Sanger sequenced in both directions using the same primers. All obtained sequences were first genotyped by comparison with the reference set provided by Valastro et al. [6]. Particularly, sequences were aligned using the MAFFT method [24] and the obtained alignment was trimmed in order to include the hypervariable region only. A phylogenetic analysis was performed using the IQ-TREE software [25], selecting as a substitution model (GTR + G [4]) the one with the lowest Akaike Information Criterion (AIC) calculated using Jmodeltest [26].

Strains clustering in the GI-19 lineage were selected and the sequencing of the full S1 gene was attempted.

To this purpose an additional external primer pair was designed using Primer3: QXS1F (5′-TGGGTGACAGTGGAAAACTG-3′) and QXS1R (5′-TGTGTTTGTATGTACTCATC-3′). The full S1 gene was thus amplified by two overlapping RT-PCR using the primer pair QXS1F-XCE2 and XCE1- QXS1R. The reaction was performed using the SuperScript ™ III One-Step RT-PCR System with Platinum™TaqDNA Polymerase kit (Thermo fisher) at the following conditions: 1X reaction mix, 0.5 μM of each primer, 1 μL of SuperScript™III RT/Platinum™Taq Mix and 5 μL of RNA template. Molecular biology grade water was added up to a final volume of 25 μL.

The cycling parameters were set at 50 °C for 30 min, 95 °C for 5 min, 45 cycles of 95 °C for 20 s and 50 °C for 20 s and 68 °C for 50 s. The presence and specificity of the PCR products was visualized on a SYBR Safe stained agarose gel. Positive samples were Sanger-sequenced in both directions using the same primer pairs.

The obtained chromatogram quality was evaluated with FinchTV (Geospiza) and consensus was obtained with CromasPro (CromasPro Version 1.5).

### Sequence analysis

All the obtained complete S1 sequences were aligned at the amino acid level and then the nucleotide sequences were back-translated using the MAFFT algorithm implemented in TranslatorX [27]. Genotyping was confirmed using the previously described approach. To evaluate the distribution of Italian GI-19 strains in the international scenario, an extensive dataset of S1 IBV sequences was downloaded from GenBank and included in the phylogenetic tree. Additionally, the presence of recombination events in the GI-19 full S1 dataset was assessed using the RDP4 software [28]. A recombination event was accepted if detected by more than two methods with a significance level lower than *p* value < 0.001 with Bonferroni correction. For each method, settings were adjusted according to the software manual. The absence of undetected recombination events was evaluated using the SBP method implemented in HyPhy [29].

The total dataset was divided in two subsets according to the vaccination protocol.

More in detail, sequences collected before GI-19 homologous vaccination introduction (i.e. November 2014) were included in the “pre-vaccination” dataset, while the remaining were in the “post-vaccination” dataset. Because of the high turnover featuring broiler farms, the new vaccination scheme was applied to all considered farms by the beginning of January 2015. No samples obtained in the intermediate period were included in the study.

Several methods, based on the ratio between synonymous (dS) and non-synonymous (dN) substitution rate estimation, were applied independently on both datasets. The presence of pervasive purifying and diversifying selection was tested using SLAC, FEL and FUBAR methods [30, 31]. Sites were considered under pervasive diversifying selection only when detected by at least two of the implemented methods, similarly to what Franzo et al. performed [17]. Episodic diversifying selection was tested with MEME [32].

The presence of episodic diversifying and directional selection was also tested on the whole dataset with FEEDS and MEDS methods, marking the post-vaccination sequences as foreground branches [33]. All analyses were performed using the multiprocessor version of HyPhy [29]. Phylogenetic trees required for the analysis were reconstructed using IQTree [25].

The significance level was set to *p*-value < 0.1 (i.e. method’s default) for the SLAC, FEL, FUBAR, FEEDS and MEDS methods, while the posterior probability threshold for the FUBAR method was set to 0.9.

### S1 subunit homology modeling

The nucleotide sequence of an Italian GI-19 strain S1 subunit was translated at amino acid level and the SWISS-MODEL web server was used to identify the best template for which quaternary structure had been experimentally determined [34]. The same program was used to estimate the protein structure through a homology-modeling approach. The obtained model was visualized and edited with Chimera [35].

## Results

### Samples and sequencing

One hundred and fifty-five samples were preliminary classified in the GI-19 lineage based on the hypervariable region analysis and were therefore included in the study. The complete S1 gene could be sequenced for 95 strains and the genotype classification was confirmed for all of them. Because prolonged circulation of live attenuated vaccines has been demonstrated in the field, the detection and sequencing of vaccine strains was considered likely. Therefore, the obtained sequences were compared with the S1 of the GI-19 based vaccines currently registered in Italy.

Four sequences, all obtained from samples collected after vaccination introduction, were removed from the dataset because of their identity with vaccine ones (strain D388).

All considered field strains formed a monophyletic group including only Italian strains (Additional file 1).

Finally, one sequence (strain 25088) was demonstrated to be a recombinant between QX and 793B strains and was also removed before further analyses. The final dataset included 90 sequences: of those, 39 and 51 were collected in the pre-vaccination and post vaccination period, respectively.

Genetic distance distribution demonstrated a substantially comparable profile between the two datasets, although the “post-vaccination” period showed a slightly higher genetic distance (Additional file 2). Therefore, the hypothesis that a higher antigenic variability may simply reflect a broader genetic heterogeneity can be disregarded. The obtained sequences have been submitted to GenBank (Acc.Numbers MK491671−MK491761).

### Selective pressure analysis: pervasive selections

Although FUBAR detected as positively selected sites codons 52, 54, 58, 65 and 132 in both datasets and 9, 64, 389 and 539 in the post-vaccination period only, pervasive diversifying selection was not detected in any site according to the decided criteria, regardless of the considered dataset.

**Figure 1:**
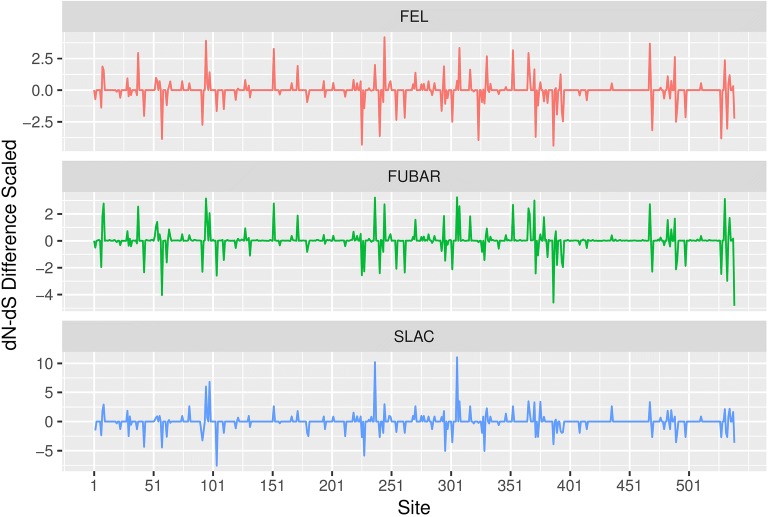
**Plot reporting the scaled dN-dS difference between the post-vaccination and pre-vaccination periods for each codon positions calculated with different methods (FEL, FUBAR and SLAC).** Positive scores are suggestive of a more intense selective pressure in the post vaccination period.

**Figure 2:**
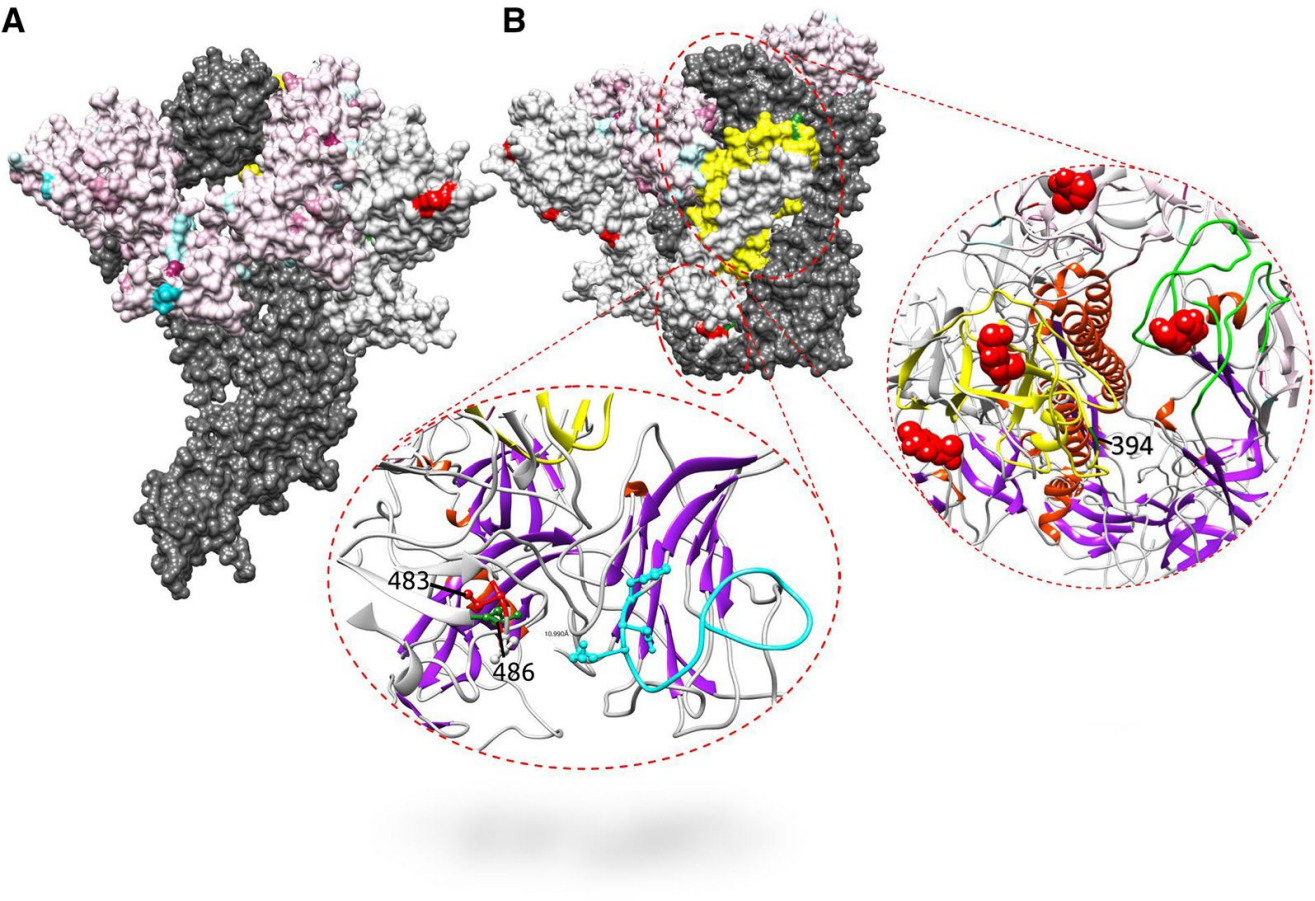
**Lateral (A) and upper (B) view of the quaternary structure of the IBV spike protein.** The full spike protein is reported for one monomer only (dark gray). The S1 region is shown for the two remaining monomers, which have been edited to highlight different selective pressure features. In the color-coded monomer, the scaled dN-dS difference between pre- and post-vaccination, calculated using FUBAR, is reported on the protein surface ranging from purple (higher pressure in the post-vaccination period) to light blue (higher pressure in the pre-vaccination period). In the white-colored monomer, sites under episodic diversifying or directional selection in the post-vaccination period are reported in red and green, respectively. The region demonstrating the higher cumulative dN-dS score (AA 300–400) is highlighted in yellow. The ribbon visualization of relevant protein domains is reported as inserts. Lower insert reports S1 NTD RBM and the partial ceiling over it (coded in light blue). Sites of the nearby monomer under episodic diversifying and directional selection in the post-vaccination period are reported in red and green, respectively. Right insert displays the S1 C-terminal domain (CTD) region. Two monomers are represented: one depicting the region with the highest cumulative score in dN-dS difference between vaccination periods (in yellow) and the other highlighting the corresponding extended putative RBM loops (in green). A more detailed representation of the overall protein structure is reported as Additional file 4.

**Figure 3:**
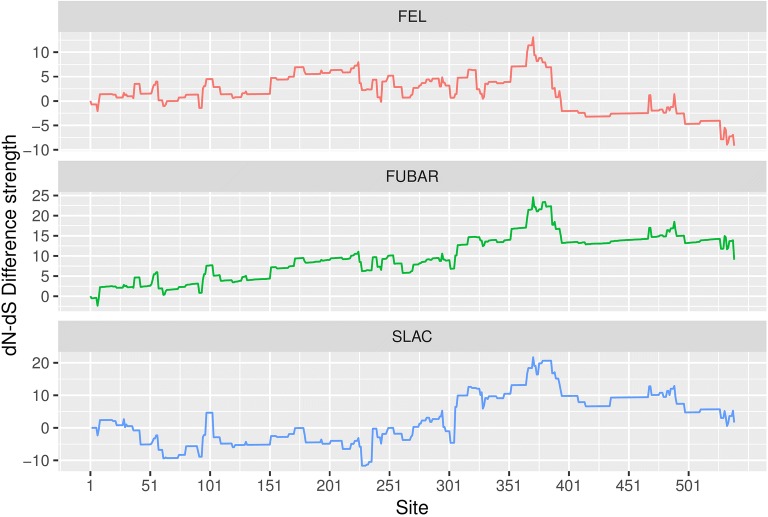
**Plot reporting the cumulative score based on the dN-dS difference between the pre- and post-vaccination periods for each codon position calculated with different methods (FEL, FUBAR and SLAC)**.

To further evaluate the presence of a differential selective pressure strength before and after the vaccination introduction, the standardized differences in dN−dS before and after vaccination introduction, estimated using FUBAR, FEL and SLAC, were calculated for each position in the alignment. Scores higher than 0, suggestive of a more prominent diversifying selection following the vaccination introduction, appeared predominant with all considered methods (Figures 1 and 2). Additionally, this value was used to calculate a cumulative score by summing it codon by codon. This score allowed to highlight the diversification tendency of S1 protein regions in the two considered time periods. Also in this case, the vast majority of S1 protein regions were characterized by higher dN−dS values in the post-vaccination introduction period, particularly in the area between aa 300 and 400 (Figures 2 and 3).

### Selective pressure analysis: episodic selections

Episodic diversifying selection could not be proven in the pre-vaccination dataset using MEME. However, positions 9, 29, 54, 65, 96 and 483 were detected in the post-vaccination dataset.

Partially comparable results were obtained using FEEDS. Codons 9, 65, 225 and 483 showed signs of diversifying selection acting on the foreground tree branches (i.e. post-vaccination period). Finally, episodic directional selection was demonstrated on 3 amino acids using MEDS, after the vaccination introduction. Particularly, a tendency to mutate toward Asparagine (N), Threonine (T) and Valine (V) was detected in positions 29, 394 and 486, respectively (Figure 2 and Additional file 3). The frequency of those amino acids rose from 0% before homologous vaccination introduction to respectively 4%, 12% and 6% after the new vaccination program implementation.

### Homology modeling

Homology modeling based on an IBV spike reference (pdb template:6CV0) [36] allowed the mapping of selective pressure sites and strength on the quaternary viral protein structure. Although minor differences between the predicted and actual QX Spike structure cannot be excluded, the good model-quality evaluation statistics provided by SWISS-MODEL support the accuracy of the obtained quaternary model.

Most of the surface amino-acids are featured by a stronger diversifying selection in the “post-vaccination” dataset (Figure 2). Particularly, the region with the higher cumulative score in dN-dS difference between pre- and post-vaccination periods corresponds to the S1 C-terminal domain (CTD) where the putative receptor-binding motif (RBM) loops are located.

The same region, exposed on the spike surface, also hosted the aa 394, detected under directional selection. Similarly, aa 486, also exposed on the spike sub-unit, is in close relationship (approximatively 10 Å) with the partial ceiling nearby another putative RBM in the S1 N-terminal domain (NTD) of the adjacent subunit (Figure 2B). The last aa detected under directional selection (aa 29) was located within the viral protein structure, instead.

All sites detected under episodic diversifying selection in the post-vaccination period are located on the spike protein surface (Figure 2).

## Discussion

Human intervention has dramatically changed the environment where pathogens evolve [18, 37]. The most evident examples are represented by the prompt emergence and spread of antimicrobial and antiviral resistance [38]. Rapidly-evolving RNA viruses seem particularly favored in the arms race against human treatments because of their ability to quickly explore the fitness landscape and new evolution pathways [39].

However, several studies have demonstrated the presence of noteworthy exceptions in veterinary medicine, most notably in intensively raised livestock like poultry and pigs [16–18]. The present work confirms this trend also for IBV, since an overall higher selective pressure strength was proven after homologous vaccination was introduced. Of note, differently from experimental infections, the molecular epidemiology-based statistical approach applied in the present study allows modelling viral evolution in its natural environment. The bias and confounding effects due to experimental conditions, animal genetic line and health status, infectious dose, selected viral strain etc. are thus avoided. Moreover, the random inclusion of several farms located in the same area before and after homologous vaccination introduction controls for the individual flock peculiarities and contingent situations, provide an overall picture of the investigated phenomenon. Several sites were detected to be under a significant episodic diversifying or directional selection in the post vaccination period only. Significantly, all the corresponding amino acids were exposed on the viral S1 surface (Figure 2), thus allowing the speculation of the role of host derived immune selection in affecting evolution.

On the contrary, pervasive diversifying selection could not be detected at the set criteria in any dataset. Based on this evidence and considering that episodic diversifying selection could be detected only in the post-vaccination dataset, an evolution occurring through a selective burst induced by homologous vaccination introduction can thus be hypothesized. Nevertheless, a lower statistical power of methods dealing only with pervasive selection could be expected [35]. When the difference between post- and pre-vaccination period in dN-dS was calculated, an overall balance in favor of a higher diversifying selection was evidenced after the homologous vaccination introduction. Therefore, although not statistically significant, a trend featured by a higher selective pressure was confirmed also by these methods (Figures 1 and 3). The highest cumulative score in dN-dS difference between vaccination periods was located in the S1 C-terminal domain (CTD), where the two extended putative receptor-binding motif (RBM) loops are located (Figure 2) [36]. This finding is particularly suggestive since antibodies raised against the RBM domain could display a higher neutralizing activity and, therefore, exert a stronger pressure toward viral diversification. Accordingly, the corresponding region (including the aa 394, detected under directional selection in the present study) was demonstrated to be involved in the selection of neutralization-resistant D274 genotype variants [40].

Similar evidence can be claimed for other sites identified in the present study, which are located within, or in close proximity to, regions like aa 24–62 and 87–93, reported as epitopes in previous experimental studies. Particularly, region 87–93 was proven to be an antigenic region specifically in the QX (GI-19) genotype [41].

Interestingly, different amino acids, i.e. aa 483 (under episodic diversifying selection) and 486 (under episodic directional selection), are located in close proximity with a loop structure constituting a partial ceiling over the S1 NTD RBM of the nearby S1 subunit (Figure 2).

Since coronavirus S1-NTDs are under the host immune pressure, the evolution of a ceiling has been suggested to provide a better protection from host immune surveillance to the sugar-binding site [39]. Antibodies binding to a nearby region could therefore decrease/inhibit the functionality of domains of pivotal relevance for viral life cycle by sterical interference. However, a potential direct role of aa 484–486 in host immune response cannot be excluded.

The congruence between the findings obtained in the present study and other experimental evidence supports the immune-induced nature of the detected pressures. Experimental confirmation could complement and confirm the results herein described, evaluating the progressive evolution of GI-19 strains in a more standardized “vaccine environment”.

The actual reasons behind the strength of selective pressures imposed by vaccination, especially in veterinary medicine, are still not fully understood.

IBV vaccine immunity is not sterilizing and a certain viral persistence in vaccinated animals is possible [42]. The scenario is further worsened by the typically partial coverage achieved by routine vaccination protocols in field conditions that, although usually effective in preventing clinical outbreaks and reducing the infectious pressure, facilitates the circulation of field viruses in a partially immunized population [21, 43]. A previous study based on a phylodynamic approach, performed in the same geographic area and timeframe, demonstrated the benefits of the homologous vaccination introduction in reducing viral population size and outbreak frequency [21]. Nevertheless, the IBV QX genotype has continued to circulate in Italy. These weaknesses can allow the presence of a viral population size high enough for natural selection to act and favor the emergence of more fit variants in a new immune-environment. Due to the early vaccination and short life span of the animals, the vaccine-induced immunity is expected to be the dominant force interacting and shaping viral evolution, rather than natural infection. Additionally, the immunity induced by a single strain (i.e. vaccine virus) is likely to be more homogeneous compared to the one elicited against heterogeneous field strains. Although the *treatment*-*mosaic*-like situation is considered one of the key factors limiting the emergence of vaccine-escape mutants, the reduced genetic variability of the poultry population imposed by commercial constraints could imply a decreased immune response spectrum, facilitating the selection of specific mutations.

Besides homologous vaccination, IBV is often controlled providing a combination of vaccines based on different genotypes (i.e. heterologous vaccination). Since a more diversified and heterogeneous immune response spectrum has been reported to arise, the emergence of specific-immunity escaping variants could be hindered.

Therefore, the benefits of heterologous versus homologous vaccination in terms of vaccine-escape induction should be carefully evaluated in the framework of the best vaccination guideline definition.

## Supplementary information

**Additional file 1. Maximum likelihood phylogenetic tree based on an extended collection of IBV S1 sequences.** The QX (GI-19) strain are highlighted ocher, while the Italian sequences have been coded in red. It is possible to zoom into appreciate further details and tip labels.

**Additional file 2. Density curves representing pairwise p-distance calculated for the pre- and post-vaccination datasets.**

**Additional file 3. Time scaled phylogenetic trees reconstructed using the QX sequences obtained in the present study**. The ancestral history of the amino acids detected under episodic directional selection has been reconstructed over time through a discrete trait analysis using the Bayesian approach implemented in BEAST1.8. Each tree has been color coded to depict the evolution of one of the considered amino acids.

**Additional file 4. Animation displaying the quaternary structure of the IBV spike protein and the action of selective pressures.**

## Data Availability

All obtained sequences have been made available in Genbank (Acc. Numbers MK491671–MK491761).
