# Supplementary figures and images for: Evolution of infectious bronchitis virus in the field after homologous vaccination introduction
Source: Vet Res. 2019 Nov 9;50:92. doi: 10.1186/s13567-019-0713-4 (PMC6842459; doi:10.1186/s13567-019-0713-4)

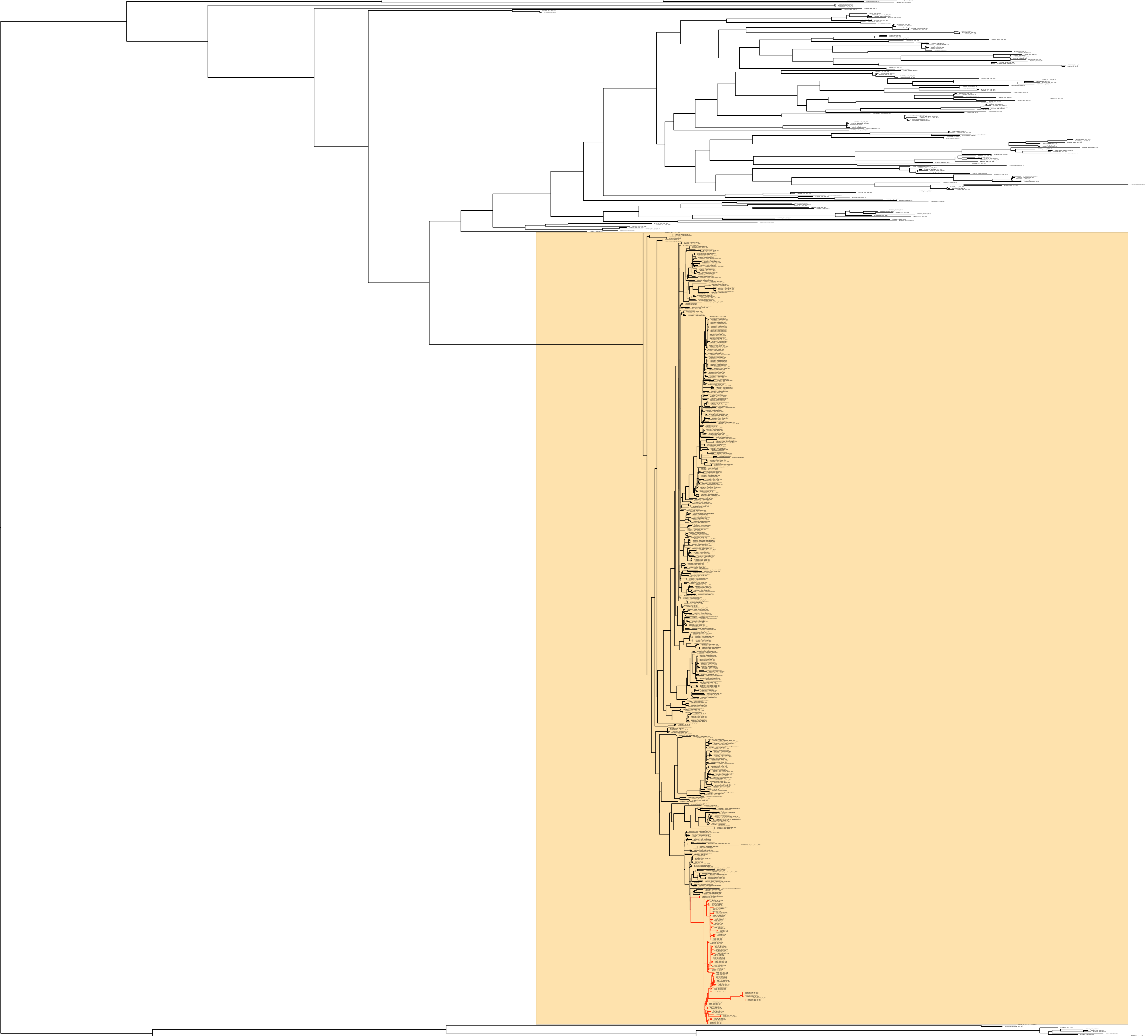

Supplement: Supplementary file 1 — Additional file 1. Maximum likelihood phylogenetic tree based on an extended collection of IBV S1 sequences. The QX (GI-19) strain are highlighted ocher, while the Italian sequences have been coded in red. It is possible to zoom into appreciate further details and tip labels. [file 13567_2019_713_MOESM1_ESM.pdf]

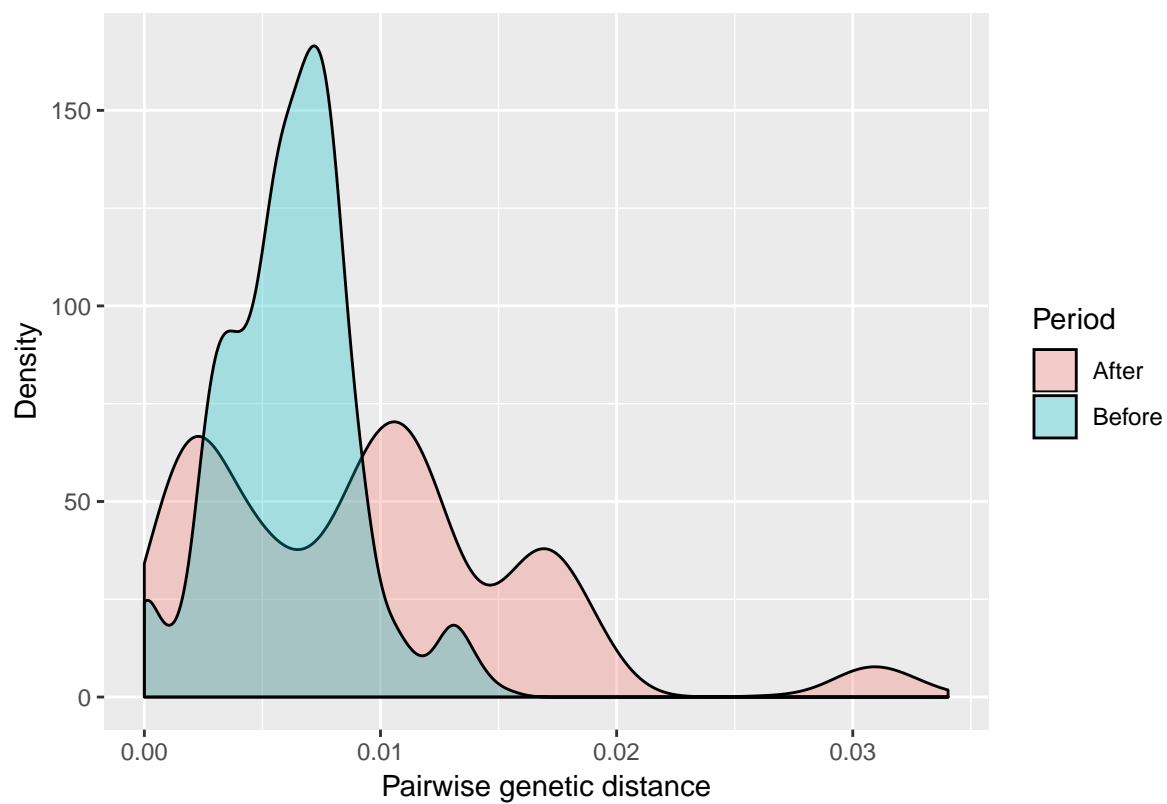

Supplement: Supplementary file 2 — Additional file 2. Density curves representing pairwise p-distance calculated for the pre- and post-vaccination datasets. [file 13567_2019_713_MOESM2_ESM.pdf]

## Amino Acid 29

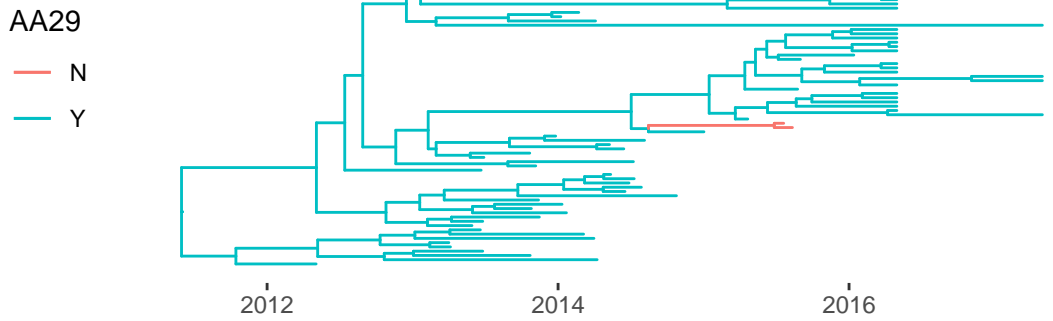

## Amino Acid 394

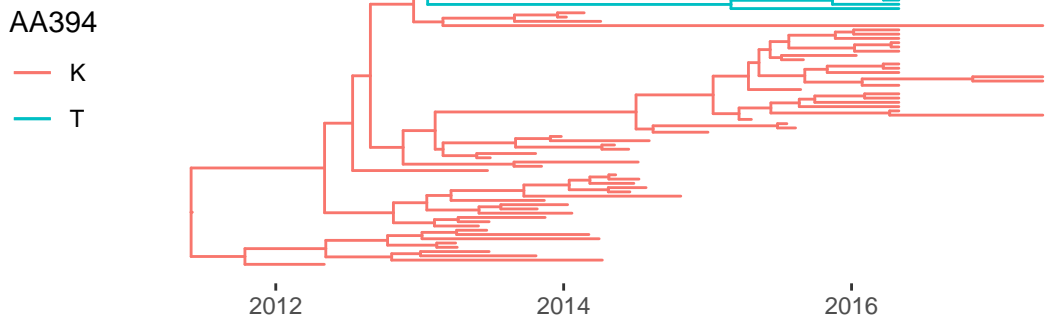

## Amino Acid 486

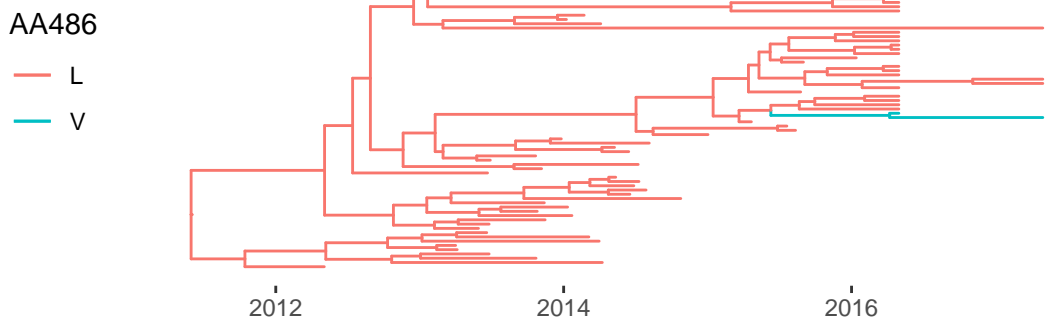

Supplement: Supplementary file 3 — Additional file 3. Time scaled phylogenetic trees reconstructed using the QX sequences obtained in the present study. The ancestral history of the amino acids detected under episodic directional selection has been reconstructed over time through a discrete trait analysis using the Bayesian approach implemented in BEAST1.8. Each tree has been color coded to depict the evolution of one of the considered amino acids. [file 13567_2019_713_MOESM3_ESM.pdf]
